# Bullous dermatomyositis with anti-NPX2 antibodies, associated with breast cancer^☆^

**DOI:** 10.1016/j.abd.2023.01.007

**Published:** 2023-12-21

**Authors:** Francisca Reculé, Juana Benedetto, Catalina Silva-Hirschberg, Raúl Cabrera, Alex Castro

**Affiliations:** a Department of Dermatology, Facultad de Medicina, Clínica Alemana, Universidad del Desarrollo, Santiago, Chile; b Department of Pathology, Facultad de Medicina, Clínica Alemana, Universidad del Desarrollo, Santiago, Chile

*Dear Editor,*

Vesicle or bulla formation in Dermatomyositis (DM) is extremely rare, with approximately 68 bullous DM (BDM) cases published to date.^1^, ^2^, ^3^, ^4^, ^5^, ^6^, ^7^ It was first reported by Christian in 1903.^8^ BDM mainly affects women in the 5^th^‒7^th^ decade. In classical DM, the male-to-female ratio is 1:2. BDM favors female presentation with a ratio of 3:1.^4^ Blisters in BDM predominantly develop on frequently irritated areas such as extensor areas of upper extremities, knees, and trunk.^4^ A recent case series addresses the importance of the correlation between BDM and neoplasia.^1^

A 54-year-old woman with a previous diagnosis of breast cancer, treated three years ago with partial mastectomy, attended our dermatology department with a three-month history of symmetric pruriginous erythematous plaques on both thighs. She was previously treated in another hospital with a 20-day course of oral prednisolone 20 mg/day with partial response. No other treatment or vaccines were prescribed. The patient progressed with generalized skin lesions within a month. Physical examination revealed an erythematous rash with symmetrical vesicles and bullas on the neck, abdomen, arms, knees, and thighs (holster sign) (Fig. 1 A and B), and Gottron’s sign (Fig. 1C). Oral mucosa was unaffected. Laboratory tests showed increased serum glutamic-oxaloacetic transaminase levels of 211 U/L, PCR 2.1 mg/dL, and total CK within normal limits. Extractable nuclear antigen, rheumatoid factor, rapid plasma reagin, anti-histone, anti-DNA, and antinuclear antibodies were negative. Complement C3 and C4 were normal. Histology demonstrated focal parakeratosis, hydropic changes in the basal layer with subepidermal edema and bulla formation. Mild lymphocytic infiltrate around superficial vessels with few neutrophils and dermal mucin deposition (Fig. 2). Direct Immunofluorescence (DIF) was only focally positive for C3 on superficial vessels. Magnetic Resonance Imaging of the extremities and neck showed mild myositis of the deltoid and trapezius muscles. The DM panel revealed positivity for anti-NPX2. A complete paraneoplastic screening was performed, which was normal.

**Figure 1:**
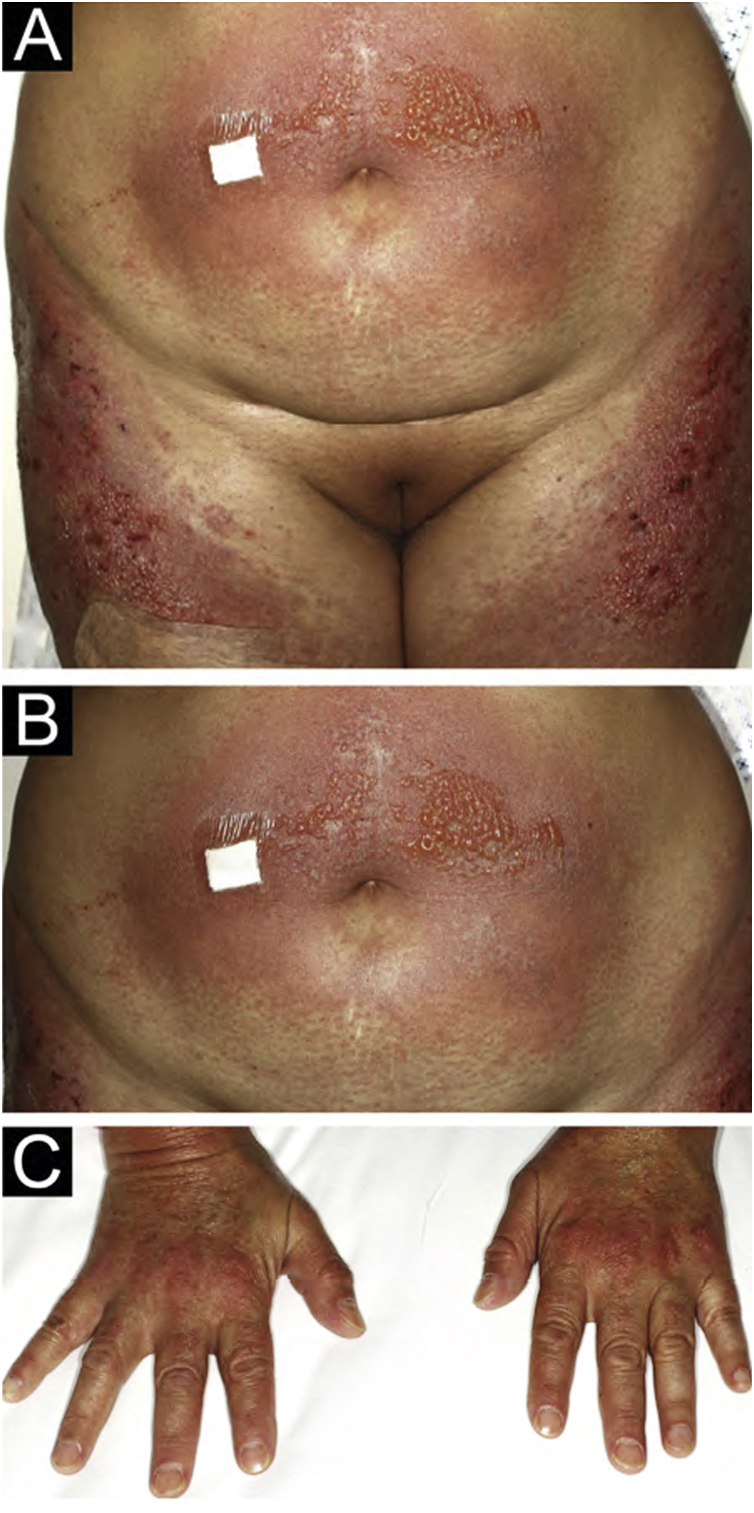
(A) Intense erythematous rash on the abdomen and thigs associated to the presence of vesicles and bullas. (B) Magnification of vesicles and bullas on the abdomen. (C) Gottron’s sign.

**Figure 2:**
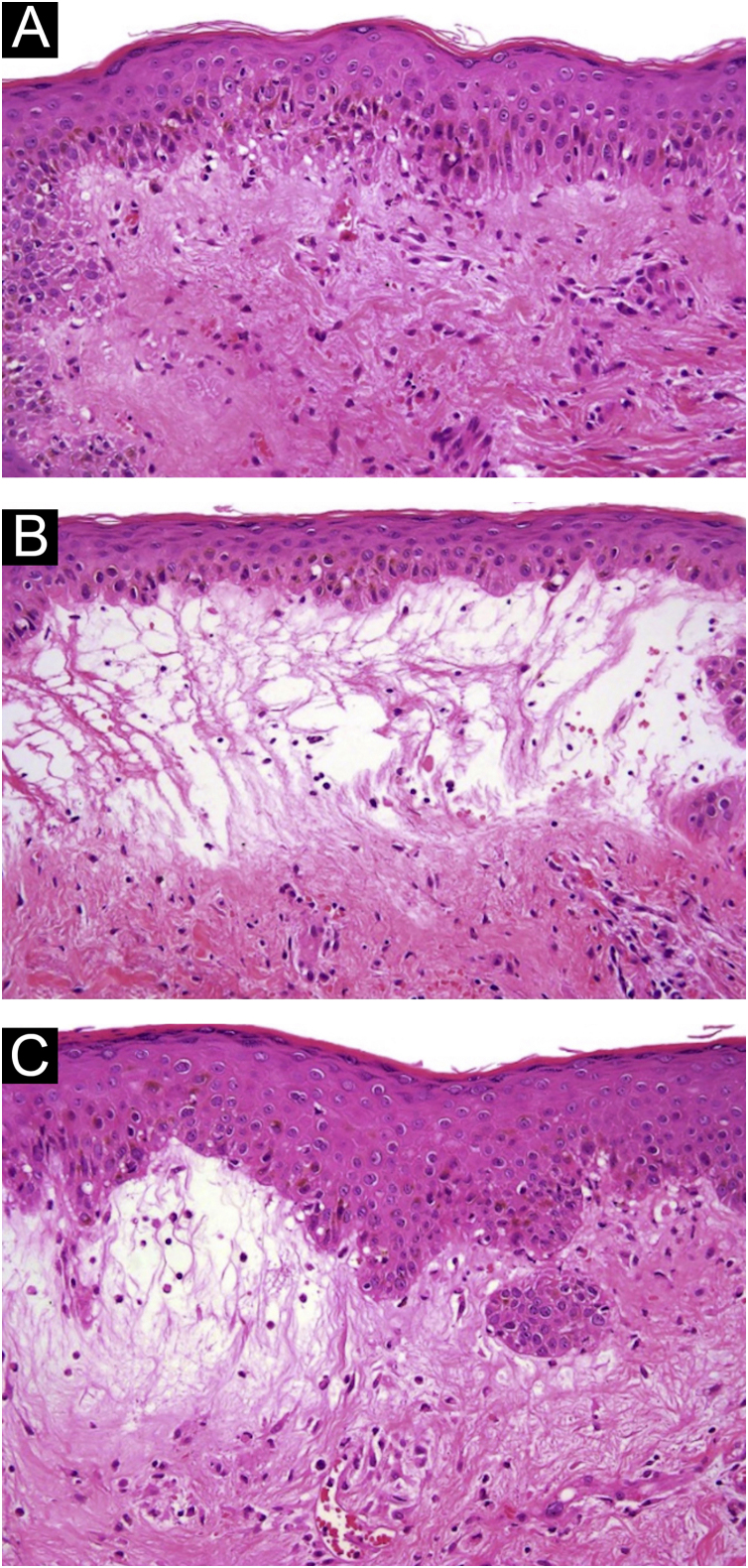
(A) 10× Hematoxylin-eosin staining; hydropic changes in the basal layer. (B and C) 10× Hematoxylin-eosin staining with marked dermal edema and bullae formation with mild lymphocytic infiltrate around superficial vessels.

Clinical and histopathological findings, in addition to anti-NPX2 positivity and the presence of vesicles lead us to the diagnosis of BDM. Treatment with oral tacrolimus 3 mg/day was administered with complete remission of blisters and subclinical myositis. Tacrolimus treatment was finished in March 2022 and, until this publication, there is no clinical recurrence. The patient has been followed for four years and no new neoplasia has been found.

Since clinical signs are usually non-specific in BDM, histopathology, and molecular markers become valuable diagnostic tools. The BDM histopathology commonly shows subepidermal blisters with dermal edema, mucin deposition, and superficial perivascular inflammation. Vesicles are generated by an intense inflammatory reaction and marked dermal edema.^4^ In about 35% of cases, DIF reveals granular deposits of immunoglobulins and complement at the dermo-epidermal junction.^9^ In addition, C3 and C5b-9 granular deposits can be found in vessels, probably as a nonspecific inflammatory reaction, supporting the diagnosis in our case.^6^, ^9^ Differential diagnoses include: (1) Bullous erythematous lupus ‒ positive granular o linear DIF in the Basal Membrane Zone (BMZ) and anti-collagen VII antibodies, (2) Bullous pemphigoid ‒ positive linear DIF in the BMZ with anti-BP180 or BP230 antibodies, (3) Dermatitis herpetiformis ‒ DIF with granular deposits in dermal papillae, and (4) Pemphigus ‒ positive intercellular DIF and anti-desmoglein 1 and 3 antibodies.^10^

DM can be associated with autoimmune blister diseases. Epitope spreading has been proposed as an explanation, but their association remains unknown.^7^

The link between DM and cancer has well known since 1960.^1^, ^6^ The association with classical DM is approximately 15%‒27%.^8^ The association between cancer and BDM is significantly higher, ranging from 52%‒68% of patients.^1^, ^3^, ^7^ The intense inflammatory reaction found in BDM is associated with a higher paraneoplastic risk.^3^, ^6^, ^7^ There is a poorer prognosis of BDM when associated with malignancies, which can develop within the first three years after BDM diagnosis.^3^ This highlights the importance of an exhaustive search for neoplasms in patients diagnosed with DM, especially if BDM is present. The finding of anti-NXP2 in DM is highly related to cancer, as it was demonstrated in this report.^2^, ^7^ BDM has also been associated with lung, ovarian, and esophagus tumors.^1^, ^3^

BDM can be easily misdiagnosed. Early recognition is key to achieve a better prognosis. Patients should be closely followed up for appropriate treatment and cancer screening. Clinical examination is mandatory every 4‒6 months for at least three years and clinical images should be performed every 6‒12 months.^3^, ^11^

Herein, we report a rare clinical presentation of hypomyopathic DM in which the combination of anamnesis, physical examination, laboratory and imaging exams, and histopathologic findings played a crucial role in reaching a correct diagnosis. We emphasize that BDM should always be in the differential diagnosis of bullous diseases.

## Financial support

None declared.

## Authors’ contributions

Francisca Reculé: Approval of the final version of the manuscript; intellectual participation in propaedeutic and/or therapeutic management of studied cases; manuscript critical review; preparation and writing of the manuscript.

Juana Benedetto: approval of the final version of the manuscript; intellectual participation in propaedeutic and/or therapeutic management of studied cases; manuscript critical review; preparation and writing of the manuscript.

Catalina Silva-Hirschberg: Approval of the final version of the manuscript; critical literature review; manuscript critical review; preparation and writing of the manuscript.

Raúl Cabrera: Approval of the final version of the manuscript; intellectual participation in propaedeutic and/or therapeutic management of studied cases; manuscript critical review; preparation and writing of the manuscript.

Alex Castro: Approval of the final version of the manuscript; manuscript critical review; preparation and writing of the manuscript.

## Conflicts of interest

None declared.
